# A Review of the Outcomes of the Implementation of Case-Based Anatomy Learning

**DOI:** 10.7759/cureus.19179

**Published:** 2021-11-01

**Authors:** Dimitrios Chytas, Vasileios Mitrousias, Vasileios Raoulis, Konstantinos Banios, Apostolos Fyllos, Aristeidis H Zibis

**Affiliations:** 1 Anatomy, University of Peloponnese, Sparta, GRC; 2 Orthopaedic Department, General University Hospital of Larissa, Larissa, GRC; 3 Emergency Department, General University Hospital of Larissa, Larissa, GRC; 4 Orthopaedics and Trauma, General Hospital of Karditsa, Karditsa, GRC; 5 Anatomy, School of Health Sciences, University of Thessaly, Larissa, GRC; 6 Orthopaedics and Musculoskeletal Trauma, University of Thessaly, Larissa, GRC

**Keywords:** review, anatomy learning, anatomy teaching, anatomy education, case-based learning

## Abstract

Purpose: Clinically-oriented anatomy education has been proposed as an effective strategy in anatomy curricula. We aimed to explore the level of extent the literature supports the fact that case-based learning (CBL) can play a significant role in anatomy education.

Materials and methods: We searched PubMed, Scopus, Education Resources Information Center (ERIC), and Cochrane database to find articles with the purpose to explore the educational outcomes of case-based anatomy learning. We extracted from each paper authors, type of study (comparative or not), number of participants, level of outcome according to the Kirkpatrick hierarchy, outcomes of CBL concerning the acquisition of anatomical knowledge, and the participants’ perceived enjoyment, motivation, and aid to anatomy learning.

Results: Nine articles were included. Three of them evaluated the acquisition of anatomical knowledge, while six papers evaluated the participants’ perceptions. All studies showed positive outcomes regarding the students’ anatomy examination performances, reported confidence, enjoyment, motivation, and ability of CBL to facilitate anatomy learning.

Conclusion: Although the existing research has mainly evaluated students’ perceptions, the outcomes of CBL in anatomy education encourage more extensive use of this method in anatomy curricula. Further research is expected to shed more light on the role that CBL can play in modern anatomy education and to clarify if it can replace or supplement didactic teaching.

## Introduction and background

Case-based learning (CBL), a well-established method in health professions education, has been defined as a “learning and teaching approach that aims to prepare students for clinical practice, through the use of authentic clinical cases” [1]. CBL is different from problem-based learning, which has been defined as "learning that results from the process of working toward the understanding of a resolution of a problem, which is encountered first in the learning process" [2]. More specifically, CBL is more structured than problem-based learning and comprises a guided inquiry method with defined learning outcomes [3]. Learners are provided with a clinical case and are given time to address it, while there is at least one facilitator who uses guiding questions to make learners focus on the main learning objective [3].

The systematic review by Thistlethwaite et al. [1] about CBL showed that it is an effective method in health professions education, probably because of its active and interactive nature. However, the authors did not focus on anatomy education and did not provide evidence that case-based anatomy learning is effective. Johnson et al. [4] proposed blended learning in a modern anatomy curriculum and emphasized the value of clinically-oriented anatomy education. Baker [5] recently confirmed that medical students are far more likely to retain anatomical knowledge related to their future clinical practice. Thus, it can be expected that CBL, which provides a clinical context to anatomy teaching, would have been accompanied by promising educational outcomes. Estai and Bunt [6], who performed a review about anatomy teaching modalities, concluded that a blended learning approach is preferable, but the review did not shed light on the role of CBL. A more recent review [7] about anatomy education strategies confirmed that blended learning could lead to the best educational outcomes, but the review did not also explore if CBL could play a role in an anatomy curriculum. Thus, we aimed to perform a review of the literature in order to explore the role of CBL in anatomy education.

## Review

Materials and methods

Three reviewers independently conducted a search in PubMed, Scopus, Education Resources Information Center (ERIC), and Cochrane database with keywords “case-based” AND “anatomy” AND (“education” OR “teaching” OR “learning”). The search was completed on March 20, 2021. To be up-to-date, we restricted our search to papers that were published in the last decade (after January 1, 2011). For the identification of relevant studies that were not detected after the initial search, the list of references of each paper included in the review was checked. The differences between the reviewers were resolved with discussion. In case of disagreement, the senior author decided.

The studies that were eligible for inclusion were those with the purpose to investigate the impact of the implementation of CBL on human gross anatomy education. Papers eligible for inclusion were those that were published in peer-reviewed journals and written in the English language. Papers without focus on CBL in anatomy education (such as papers that explored education of anatomy in conjunction with other disciplines, without distinct outcomes), duplicates, review articles, letters to the editor, expert opinion articles, and conference papers were excluded. The screening took place in three stages and included title, abstract, and full text. If the title did not clarify whether the study would be included or excluded, the abstract was also screened. Finally, if the abstract did not provide such clarification again, the full text was screened. The following data were received from each paper: authors, type of study (comparative or not), number of individuals who participated, level of outcome according to the Kirkpatrick hierarchy [8,9] (Table 1), outcomes of the implementation of CBL concerning the acquisition of anatomical knowledge, and participants’ perceptions about the extent to which CBL provided enjoyment, motivation, and aid to anatomy learning.

**Table 1:** Kirkpatrick hierarchy [8,9]

Level 1	Reaction	Relates to participants’ opinions on the learning experience
Level 2a	Change of attitudes and perceptions	Relates to changes in the participants’ attitudes or perceptions after the educational intervention
Level 2b	Change of knowledge and skills	Relates to the acquisition of knowledge and skills after the educational intervention
Level 3	Behavioral change	Relates to the change of behavior in the workplace due to the educational intervention
Level 4a	Change in organizational practice	Significant changes in the delivery of care due to an educational program
Level 4b	Benefits to patients	Improvement of patients’ health due to an educational program

Results

In total, 83 articles were retrieved after the initial search. We excluded 20 articles that were without focus on the implementation of CBL in anatomy education, six reviews, two conference papers, and 46 irrelevant articles. Thus, nine articles were included [10-18] (Table 2).

**Table 2:** Basic characteristics of the studies of the review (authors, number of participants, type of study, level in the Kirkpatrick hierarchy)

Authors	Number of participants	Type	Level
Demetri et al. [10]	35	Comparative	2b
Holland and Pawlikowska [11]	164	Comparative	1
Gheysens et al. [12]	28	Comparative	1
Parmar and Rathinam [13]	20	Comparative	2b
Eseonu et al. [14]	43	Comparative	2b
Sinha et al. [15]	164	Non-comparative	1
Singh and Bhatt [16]	83	Non-comparative	1
Jarral et al. [17]	115	Non-comparative	1
Uma et al. [18]	123	Non-comparative	1

There were five comparative [10-14] and four non-comparative studies [15-18]. There were three studies [10,13,14]that evaluated the participants’ anatomy examinations performance after the implementation of CBL; thus, they had a level 2b in the Kirkpatrick hierarchy. Six studies [11,12,15-18] evaluated only the participants’ perceptions about CBL; thus, they had a level 1 in the Kirkpatrick hierarchy.

Comparative Studies About Case-Based Anatomy Learning

Demetri et al. [10] developed CBL sessions to teach in an orthopedic resident anatomy course. The study involved 35 participants who were taught a specific content via lectures and a different content via CBL. Separate examination results were evaluated for the content, which was taught via CBL and lectures. At the end of the course, insignificant differences between the two modalities were noted regarding the examination performances (84% versus 78%), while CBL sessions were perceived as very helpful, in terms of enhancing anatomy knowledge, but with an insignificant difference from lectures.

The questionnaire study by Holland and Pawlikowska [11] comprised 164 medical students, who received case-based anatomy lessons that are available to students in their anatomy course books and also online. Over half of the participants accessed at least one online case, while 18% accessed all eight online cases that were available. The students were asked to compare the online format with the format comprising small group tutorial (anatomy room) discussion. Both formats were rated highly not only with an insignificant difference in terms of facilitating anatomy learning (3.97 versus 3.75, respectively, in a five-point Likert scale) but with significant superiority of the online format in terms of enjoyment (3.66 versus 3.34 in a five-point Likert scale).

Gheysens et al. [12] created an interactive, case-based module to teach spinal cord anatomy. The first version of the module included a review of spinal cord anatomy followed by clinical cases with questions. Students were prompted to draw pictures depicting spinal cord anatomy. The second version included the same materials, while the students reviewed premade images and did not draw. Twenty-eight participants were randomly assigned in two equal groups to receive neuroanatomy teaching with either the first or second module. According to the students’ answers on a five-point Likert scale, their confidence to describe spinal cord anatomy improved significantly, while the difference between the two module versions was insignificant. Students graded satisfaction with the module as 3.8/5, with slightly higher satisfaction in the group that completed the first version.

Parmar and Rathinam [13] applied CBL in the anatomy education of 20 students of occupational and physical therapies. The participants’ anatomical knowledge was assessed before the intervention as well as immediately after it and four weeks later. Significant improvement in the students’ examinations performance was noted between pre-intervention and post-intervention scores, both immediately and after four weeks. Almost all participants felt that CBL enhanced their learning of anatomy.

Eseonu et al. [14] included in their study 43 clinical-phase medical students who received case-based anatomy teaching. They had already been taught anatomy in the preclinical phase of their curriculum via other methods. Before the educational intervention, they self-rated their confidence in anatomical knowledge and undertook a test. Four weeks after the educational intervention, students’ anatomical knowledge and reported confidence were significantly enhanced.

Non-comparative Studies About Case-Based Anatomy Learning

Sinha et al. [15] performed a questionnaire-based study that explored the perceptions of 164 medical students about the incorporation of CBL into their anatomy curriculum. The sessions emphasized the clinical importance of anatomical details, providing students with multiple examples of how the anatomy knowledge they acquire is transferred in clinical practice. Each patient challenge had a solution deriving from a mastery of anatomy, and all participants were encouraged to suggest solutions in an open forum format to the patient challenges that were encountered. Eighty-six percent of the participants stated that CBL increased their mastery of human anatomy, while 77% believed that CBL increased their examination performance.

Singh and Bhatt [16] taught anatomy to medical students via CBL, which consisted of two sessions. The first session included a small group discussion among the students who were helped by a facilitator. The role of the facilitator was to observe and keep the discussion on the right track. In the second session, the groups presented their discussion outcomes. Eighty-three students provided feedback, and 62% of them stated that CBL was useful for promoting understanding of anatomy, while 14% disagreed and 24% had a neutral opinion.

The study by Jarral et al. [17] included 115 medical students, who were taught anatomy via CBL. Students, divided in small groups, were provided with a PowerPoint presentation (Microsoft Corporation, New Mexico, USA) about the clinical case and participated in the discussion section, while the teacher had the role of facilitator and summed up the case at the end of the session. About nine out of 10 participants stated that CBL was useful in terms of enhancing their anatomy knowledge. About 84% of the respondents stated that CBL was more effective than didactic lectures; the same percentage felt happy about the learning experience, while about seven out of 10 students argued that CBL motivated them to study anatomy.

The study by Uma et al. [18] included 123 medical students who received case-based anatomy learning during the appropriate cadaveric dissection session. The case scenarios were followed by questions on the anatomy related to the case, and by a discussion facilitated by a faculty member. Almost all students stated that CBL was helpful for enhancing their understanding of anatomy, while 95% of them argued that this method increased their motivation to study.

Discussion

All nine studies of the current review indicate that CBL could have a remarkable value as an anatomy education tool. However, the research regarding CBL in anatomy education is relatively limited, is mainly focused on the evaluation of participants’ perceptions, and does not include many studies that evaluated the acquisition of anatomical knowledge. Also, most studies that explored either respondents’ perceptions or examinations performance did not comprise comparison with other anatomy education methods. It seems that CBL has notable anatomy teaching potential, which needs further investigation with comparative studies. Further investigation will also clarify to what extent the effectiveness of CBL is associated with specific factors such as the students' and teachers' competency and the quality of case-based teaching.

Students’ Examinations Performance After Case-Based Anatomy Learning

Three studies [10,13,14] investigated if CBL was effective in terms of the acquisition of anatomical knowledge. Demetri et al. [10] comprised orthopedic residents in their study, while Eseonu et al. [14] as well as Parmar and Rathinam [13] comprised students. Demetri et al. [10], who compared CBL with lectures, found that both methods led to high grades and were equally effective for residents. However, an important limitation of this study is the lack of evaluation of participants’ knowledge before the educational intervention. Thus, it could not be clarified if CBL led to significant improvement of participants’ examinations performance. Also, it can be pointed out that orthopedic residents have better familiarity with clinically applied anatomy in comparison with medical students. This increased familiarity may have played a role in reducing the educational potential of CBL regarding the acquisition of anatomical knowledge.

In contrast with the studies by Demetri et al. [10], Eseonu et al. [14], as well as Parmar and Rathinam [13], students compared their examinations performance before and after the educational intervention and found that CBL led to significant improvement. Of note, both studies evaluated anatomical knowledge four weeks after the teaching sessions, while Parmar and Rathinam [13] evaluated the performance of the examinations immediately after CBL. This was the only study that investigated not only the acquisition but also the retention of anatomical knowledge and found that four weeks after the intervention, a significant improvement of knowledge remained. However, it can be noted that Eseonu et al. [14] as well as Parmar and Rathinam [13] did not compare CBL with other anatomy teaching modalities. It would be useful if such comparative studies were performed in the context of undergraduate and postgraduate anatomy education in order to clarify if CBL is more effective than other approaches. Overall, it can be pointed out that CBL has the potential to promote anatomical knowledge gain and retention. This potential could encourage more extensive implementation of CBL in anatomy curricula and the performance of more comparative studies, which will further investigate if CBL can replace or supplement didactic anatomy teaching.

Perceptions About Case-Based Anatomy Learning

All studies of the current review included evaluation of students’ perceptions about CBL. All articles showed that these perceptions were positive, concerning all the investigated parameters. In all included papers that explored students’ perceived effectiveness [10,11,13,15-18], the participants generally agreed that this method was helpful in terms of enhancing their anatomical knowledge. In the study by Sinha et al. [15], almost eight out of 10 respondents stated that CBL enhanced their examinations performance, although the study did not explore gain of knowledge. In the study by Jarral et al. [17], the vast majority of the respondents stated that CBL was the more effective anatomy teaching method than lectures. However, this paper investigated the implementation of only CBL and not lectures in anatomy education. It is evident that the participants evaluated their experience with didactic anatomy education prior to receiving CBL. Of note, Gheysens et al. [12] compared students’ perceptions about the educational value of two different modules of CBL with or without drawing activity and found an insignificant difference. Holland and Pawlikowska [11] also compared two modules of CBL, online and anatomy room discussion, and also found an insignificant difference in terms of perceived effectiveness. There was no other study in the review to compare different modules of CBL. From the two aforementioned studies, safe conclusions about the best way of delivery of case-based anatomy education cannot be extracted.

Overall, the current review showed that CBL was a highly appreciated method, in terms of motivating students, promoting their confidence, and creating satisfaction. Jarral et al. [17] and Uma et al. [18] explored if CBL motivated students to learn anatomy and found that the vast majority of participants argued that their motivation was enhanced. Gheysens et al. [12] and Eseonu et al. [14], who compared students’ reported confidence before and after CBL, noted that it increased significantly after CBL. Holland and Pawlikowska [11], Gheysens et al. [12], and Jarral et al. [17] investigated if CBL led to students’ satisfaction and found that a very high percentage of participants stated happiness after the implementation of this method. These encouraging results could signpost that CBL may lead to increased effectiveness of anatomy education and be more extensively applied in anatomy curricula. Further research, including comparative studies, is needed to confirm these positive outcomes.

Limitations

The current review has some limitations. First, the search in the specific databases with our keywords, inclusion and exclusion criteria, may have not been able to detect all the papers that investigated the educational outcomes of case-based anatomy learning. Furthermore, the number of articles included in the review could not be considered as high. If more than nine papers had been included in the review and if it was systematic with meta-analysis, the value of CBL would have been more thoroughly investigated. Performance of systematic reviews with meta-analysis on this topic in the future will further clarify the role of case-based anatomy learning.

## Conclusions

The outcomes of the application of CBL in anatomy education are encouraging. However, the research so far has mainly focused on students’ perceptions, which indicate that CBL is a highly acceptable method. The relatively limited data about the effectiveness of CBL in terms of anatomical knowledge gain are also positive and indicate that the implementation of CBL in anatomy curricula should be encouraged. There is currently inadequate evidence to clarify if CBL can replace or supplement didactic teaching. Ongoing research is expected to shed light on the role that CBL can play in modern anatomy education and in the blended learning and clinically-oriented approach that has been proposed.
